# Efficient Purification of Auto-Exhaust Soot Particles Using Hexagonal Fe_2_O_3_ Nanosheets Decorated with Non-Noble Metals (Ni)

**DOI:** 10.3390/nano15030233

**Published:** 2025-02-01

**Authors:** Haoqi Guo, Jing Xiong, Peng Zhang, Jian Liu, Zhen Zhao, Yuechang Wei

**Affiliations:** 1 State Key Laboratory of Heavy Oil Processing, College of Science, China University of Petroleum, Beijing 102249, China; 2022211354@student.cup.edu.cn (H.G.); 2019310807@student.cup.edu.cn (P.Z.); liujian@cup.edu.cn (J.L.); zhenzhao@cup.edu.cn (Z.Z.); 2 Key Laboratory of Optical Detection Technology for Oil and Gas, China University of Petroleum, Beijing 102249, China

**Keywords:** Fe_2_O_3_ nanosheet, cooperative effect, heteroatomic catalyst, soot purification

## Abstract

Purification of soot particles from automobile exhaust has closely to do with the synergistic effect between catalyst metals. Here, several binary Ni-Fe oxide catalysts were elaborately prepared via a modified solvothermal method. A non-noble-metal (Ni)-modified hexagonal Fe_2_O_3_ nano-sheet catalyst (Ni−Fe_2_O_3_) was prepared. The introduced heteroatoms replace some of the Fe atoms, which take up the surface of the [FeO_6_] octahedron, and the synergistic effect formed between the heteroatoms which are on the surface and the adjacent Fe atoms promotes the formation of coordination unsaturated ions of the activated reactants. The optimal performance was obtained with the Ni-Fe_2_O_3_-20 composition, with catalytic soot oxidation resulting in T_50_, SCO_2_^m^, E_a_ and TOF of 366 °C, 99.1%, 72.7 kJ mol^−1^ and 0.156 min^−1^ (at 310 °C), respectively. The combination of Ni and Fe_2_O_3_ cells increases the ratio of Fe^3+^/Fe^2+^, making the interaction among electrons between the Ni, which was proved highly dispersed over the catalyst, and the Fe_2_O_3_ strong. Both exist on the catalyst surface in the form of NiFe_2_O_4_. Ni atoms and Fe_2_O_3_, which demonstrate a synergistic effect, promoting the formation of coordination unsaturated ions of the activated reactants and generating more oxygen vacancies, thus promoting the adsorption of NO and accelerating the ignition of soot in O_2_ at a low temperature. The novel Ni-Fe_2_O_3_-X oxide cocatalyst is an improved noble-free catalyst that promotes the synergistic effect between heteroatoms and metal oxides through surface regulation. This is of great significance for the further development of economic and efficient catalysts for soot particle removal from automobile exhaust.

## 1. Introduction

Most cities in the world are suffering from the effects of environmental pollution, especially those in developing countries. Among all the problems of environmental pollution, the harm caused by haze has come into the public’s vision in recent years. This pollution mainly comes from small particles in the air. Of all particulate pollutants, particles smaller than 2.5 μm (aerodynamic diameter as the standard) in diameter have the greatest impact on people. On the one hand, haze will reduce the visibility of the environment and cause traffic accidents; on the other hand, smog also seriously affects human life and can cause health problems, which is a security hazard that we cannot ignore. Recent studies have found that diesel exhaust accounts for a large proportion of particulate matter (PM) pollution in China. In view of this situation, it is particularly necessary to regulate the emissions of these pollutants mentioned above in order to minimize the impact on all aspects of human life [1]. For decades, the current level of technology still makes us have to rely on the introduction of precious metals into the catalyst to achieve the purpose of cleaning the exhaust gas. The use of these precious metals to support platinum-group metals (PGMs) on catalysts is most common. However, the limitations of precious metals force us to further research the application of catalysis in automobiles. In other words, the development of safe, low-cost, easy to access catalysts can replace precious metals is an urgent need for such large-scale deployment [2].

Catalytic diesel particulate filter (CDPF) technology stands out among many approaches to the problem of PM emissions. It is recognized as by far the most effective technology for reducing PM emissions. The key to this technology lies in the research of catalyst. Because the temperature window of diesel exhaust gas is (200 ~ 500 °C), the catalyst developed by CDPF can completely oxidize soot, which can only be oxidized at high temperature, in this temperature window, so as to achieve the purpose of efficient cleaning. At present, catalysts based on noble metals are widely regarded as efficacious catalysts for diesel soot oxidation. However, the high cost and difficulty of obtaining noble metals limit their application. Non-noble metals, such as alkaline metal oxides [3], transition metal oxides [4], perovskite-like oxides [5] and ceria-based oxides [6], have been investigated. It is found that they have good catalytic performance for soot combustion, which is not weaker than that of noble metal catalysts. Among them, the catalyst with Fe_2_O_3_ as the base stands out.

In recent years, Fe-based catalysts have been studied, including Pt/Fe_2_O_3_ [7] and Fe_2_O_3_@CeO_2_ [8], in which noble metals and rare earth metals are inevitably used. This has caused a lot of trouble in terms of raw material access and economics. In the process of soot catalytic oxidation, although SnO_2_ and Cr2O_3_ catalysts plays a significant role in the adsorption of NO [9], and promote the adsorption of CO through pre-adsorbed NO, SnO_2_ is similarly more inclined to support precious metals (such as Pt, Ce) as catalysts [10]. For Mn-Fe_2_O_3_ catalysts [11], although the cost problem has been solved, the performance of catalytic oxidation of soot needs to be improved. Based on the existing research results of different researchers, non-noble-metal catalyst (Ni-Fe_2_O_3_-X)-modified hexagonal Fe_2_O_3_ nanosheets were successfully synthesized by the hydrothermal method [12] in this paper. Considering all aspects of soot removal performance of chemical agents, the catalyst formed by introducing heteroatom Ni into Fe_2_O_3_ has greatly adsorbed and activated NO and O_2_, and thus greatly improved the soot removal performance [13]. Incorporated with the characterization results, the synergistic effect and catalytic mechanism of Ni-Fe_2_O_3_-X are described in detail, which provide a basis for the further progress of efficacious and inexpensive and clean catalysts for the elimination of automobile exhaust soot particles [14].

## 2. Materials and Methods

### 2.1. Synthesis of Fe_2_O_3_ and Ni-Fe_2_O_3_-X Oxide Systems

The Ni-Fe_2_O_3_-X nanosheet as the catalyst precursor was prepared by a hydrothermal method. The synthesis processes and the details are described as follows: Fe (NO_3_)_3_· 9H_2_O and Ni (NO_3_)_2_·6H_2_O (amounting to 5 mmol) are dissolved in 60 mL deionized water and strongly stirred for 10 min until the solution becomes a transparent and uniform clarified solution. The composition of different catalysts is shown in Table 1. After obtaining a clarified solution, 1.6 mL ammonium hydroxide solution is slowly added to the solution and stirred thoroughly until there is no more precipitation. Then, the solid–liquid mixture described above is transferred into an autoclave (100 mL). The heating time is 12 h, and the heating temperature is 180 °C. After the mixture cools naturally, it is centrifuged and collected. The collected sediment is washed 3 times. The solvent used is deionized water. After that, the solid is washed once with centrifugal ethanol and dried in a vacuum for about 24 h. The precursor of Ni-Fe_2_O_3_-X is procured. Finally, before the precursor was ready for use, it needs to be calcined. The calcination temperature is 500 °C and the calcination time is about 4 h. The product is named as the Ni-Fe_2_O_3_-X catalyst (X refers to the total molar content of Ni in all metals).

### 2.2. Materials Characterization

The details of the characterization methods are shown in the Supporting Information.

### 2.3. Catalytic Performance Evaluation

The catalytic activity of Ni-Fe_2_O_3_-X catalyst for temperature programmed oxidation (TPO) of soot combustion in a fixed bed tubular quartz system was studied. The initial temperature of each test is 150 °C, the final temperature is 600 °C, and the warming rate is 2 °C min^−1^. The soot particles are commercial soot particles purchased from Degusse (Printex-U). The soot particles are used to replace the solid components in soot exhaust. The catalyst amounting to 100 mg and soot amounting to 10 mg are added and evenly mixed in an agate bowl. The contact mode of the two is loose contact. The details can be found in the Supporting Information.

## 3. Results

### 3.1. XRD Analyses

**Figure 1:**
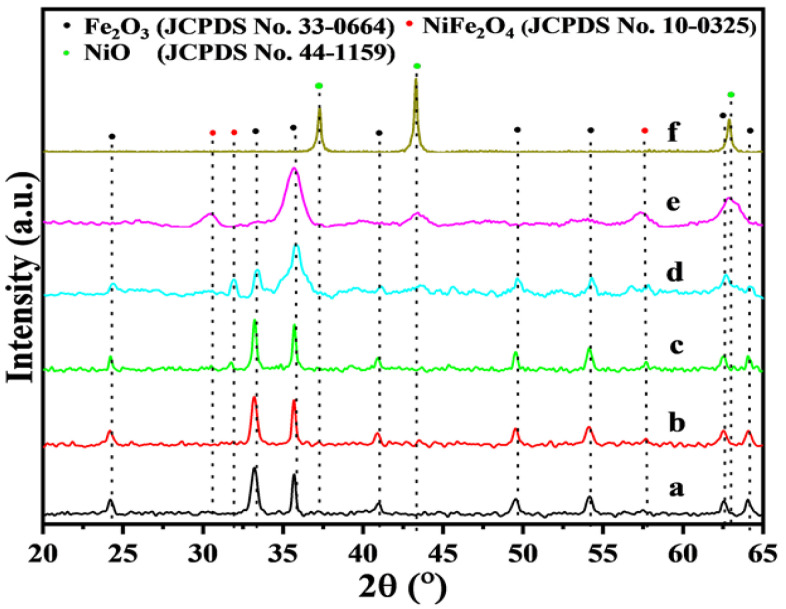
XRD patterns of Fe_2_O_3_ and Ni−Fe_2_O_3_-X catalysts: (a) Fe_2_O_3_, (b) Ni-Fe_2_O_3_-5, (c) Ni-Fe_2_O_3_-10, (d) Ni-Fe_2_O_3_-20, (e) Ni-Fe_2_O_3_-30 and (f) NiO.

The phase structure is an essential part of understanding the catalyst and can be visually understood through XRD patterns. Figure 1 illustrates nine main peaks in the position of 24.1, 33.2, 35.6, 40.9, 49.5, 54.1, 57.6, 62.4 and 64.0^o^ belonging to the cubic phase Fe_2_O_3_ (JCPDS: 33-0664) [15]. For Ni-Fe_2_O_3_-X (X refers to the total molar content of Ni in all metals) catalysts, in addition to the original diffraction peak of Fe_2_O_3_, three diffraction peaks at 30.3, 35.7 and 62.9^o^ were observed, belonging to the (220), (311)and (440) faces of trevorite NiFe_2_O_4_ (JCPDS: 10-0325), respectively [16]. In comparison with the weak diffraction peaks of pure NiO (JCPDS: 44-1159) and Ni_2_O_3_ (JCPDS: 14-0481), no diffraction peak was observed in the Ni-Fe_2_O_3_ catalyst, indicating that although the content of Ni atoms increased, it was not enriched in the form of NiO and Ni_2_O_3_ on the surface of the Fe_2_O_3_ nanosheets. In other words, the crystallinity of the catalyst decreased as the amount of Ni atoms introduced gradually increased, indicating that Ni was successfully introduced into the lattice of Fe_2_O_3_, replacing the Fe atoms in the hematite structure [17] and the NiFe_2_O_4_ structure was formed on the surface of the catalyst. For catalyst Ni-Fe_2_O_3_-20, as shown in Figure S4, XRD before and after the reaction shows that the remaining positions remain unchanged, although the intensity of some peaks decreases, indicating that the phase structure of the catalyst before and after the reaction does not alter basically. This phenomenon also reflects the stability of the catalyst [18].

### 3.2. Raman Spectra

**Figure 2:**
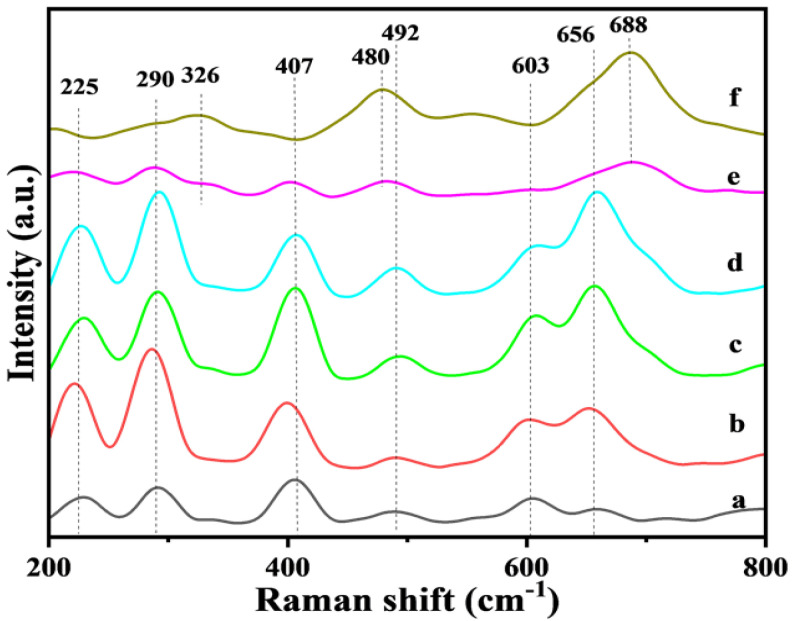
Raman patterns of Fe_2_O_3_ and Ni−Fe_2_O_3_-X catalysts: (a) Fe_2_O_3_, (b) Ni-Fe_2_O_3_-5, (c) Ni-Fe_2_O_3_-10, (d) Ni-Fe_2_O_3_-20, (e) Ni-Fe_2_O_3_-30 and (f) NiO.

In the study of the catalyst’s basic structure, Raman spectroscopy is able to be used to identify the prepared Fe_2_O_3_ and Ni-Fe_2_O_3_-X catalysts under visible light irradiation at 532 nm wavelength [19], and the results are illustrated in Figure 2. The prepared catalyst has an distinguishable Raman peak centered at 138 cm^−1^, which can be considered to correspond to the vibration of the Fe-O bond [20]. Comparing the curves corresponding to the four catalysts (from a to d), it can be seen that no other distinguishable Raman peaks appeared after the introduction of Ni, and these Raman peaks all show an obvious blue shift, indicating the successful introduction of Ni [21]. In the Raman spectrum, the original Ni-Fe spinel shows five identifiable Raman bands, i.e., where the numbers and dotted lines were marked in the figure, working in concert with the E_g_ (326 cm^−1^), A_1g_ (688 cm^−1^) and T_2g_ (225, 480, 603 cm^−1^) [22] vibration modes, indicating the existence of a NiFe_2_O_4_ spinel structure. The intensity of these Raman peaks increases gradually with the increase in Ni content. The results correspond to the presence of NiFe_2_O_4_ crystal faces in XRD, thus proving the existence of an Fe-Ni cooperative effect [23].

### 3.3. TEM and EDS Mapping Images

**Figure 3:**
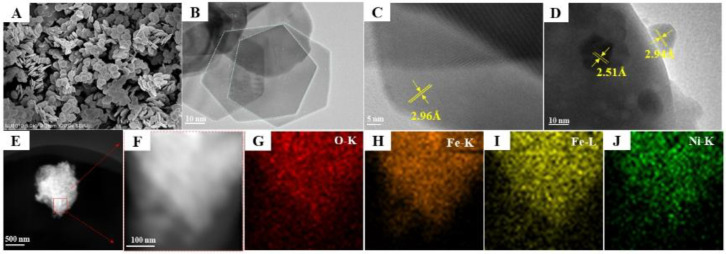
(**A**–**D**) TEM and (**E**–**J**) EDS mapping images of Ni-Fe_2_O_3_-20 catalyst.

The catalytic performance in the heterogeneous catalysis process is mainly determined by the morphology of the catalyst and the exposed surface [24]. This property can be measured by TEM techniques. Correlation results are displayed in Figure 3A,B. Pure Fe_2_O_3_ presents a polyhedral structure, the side length of which is in the vicinity of 100–150 nm [25]. After introducing heteroatoms (Ni), the polyhedral structure turns into the nanoparticle shape. This phenomenon is consistent with the decrease in crystallinity obtained by XRD. Ni affects the growth of Fe_2_O_3_ and indirectly promotes the formation of NiFe_2_O_4_ on the surface [26]. As shown in Figure 3C, the lattice spacing is 2.96Å, and the exposed facet on the side of the structure is the {110} facet of the Fe_2_O_3_ polyhedron. After introducing Ni, as shown in Figure 3D, lattice fringes with spacing of 2.51Å and 2.96Å are revealed, consistent with the (220) plane of NiFe_2_O_4_. In addition, EDS elemental mapping is used to study the distribution of elements. In Figure 3G–J, the results are shown. It can be seen that O represented by red, Fe-K represented by orange, Ni represented by green and Fe-L represented by yellow are evenly distributed throughout the image, indicating that Ni is successfully and evenly dispersed in the Fe_2_O_3_ nanoparticles. In summary, a Ni-Fe_2_O_3_ catalyst with Fe atoms uniformly replaced by heteroatoms (Ni) in Fe_2_O_3_ was successfully prepared.

### 3.4. The Results of XPS Spectra

**Figure 4:**
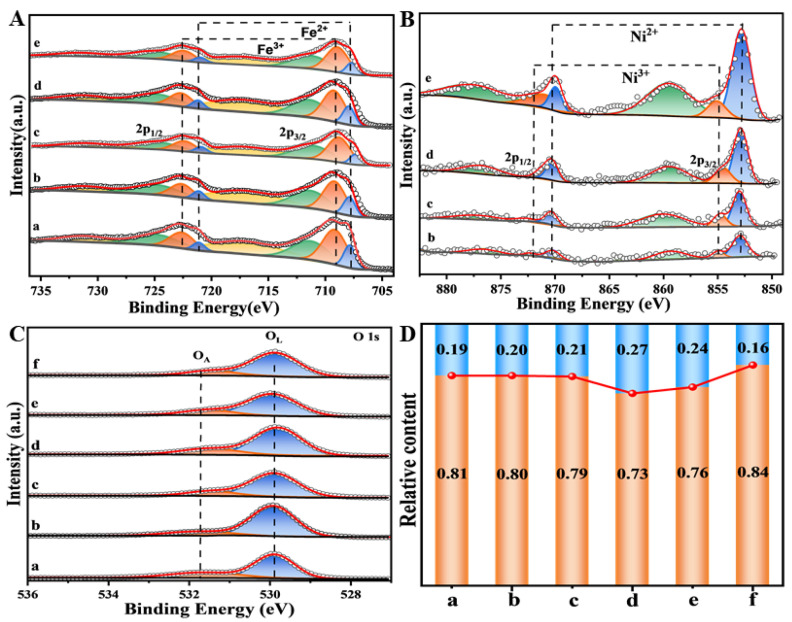
(**A**) Fe 2p spectra of Fe_2_O_3_ and Ni−Fe_2_O_3_-X catalysts: (a) Fe_2_O_3_, (b) Ni-Fe_2_O_3_-5, (c) Ni-Fe_2_O_3_-10, (d) Ni-Fe_2_O_3_-20, (e) Ni-Fe_2_O_3_-30, (**B**) Ni 2p spectra of Ni−Fe_2_O_3_-X catalysts, (**C**) O_A_ and O_L_ content of Fe_2_O_3_ and Ni−Fe_2_O_3_-X catalysts, (**D**) O_A_ (blue) and O_L_ (orange) content histogram of Fe_2_O_3_ and Ni−Fe_2_O_3_-X catalysts.

The surface elemental valence state and composition were measured by XPS measurements. It is of significance for the catalytic activity of heterogeneous catalysis [27]. The resulting Fe 2p, Ni 2p and O 1s spectra are illustrated in Figure 4. As shown in Figure 4A, Fe^2+^ (707.7, 721.1 eV) and Fe^3+^ (709.1 and 722.6 eV) of Fe ions are present on the surface of Fe_2_O_3_ and Ni-Fe_2_O_3_-X samples after deconvolving, and their peaks are filled with blue and orange, respectively. Additionally, as shown in Figure 4B and Figure S5, Ni-Fe_2_O_3_-X catalysts exhibit four main peaks at 852.9, 870.3, 855.0 and 872.2 eV, which are attributed to the Ni 2p_1/2_ and 2p_3/2_ spin orbits, respectively [28]. In addition, the presence of two strong Ni^2+^ satellites (859.3 eV and 876.9 eV) in the Ni 2p spectrum indicates that the principal valence state of Ni is +2. Table S2 summarizes the containing of Fe^2+^ and Fe^3+^ and the relative ratio of Fe^2+^ to Fe^3+^ (Ra). Compared with all samples, the Fe^3+^ ion is the main type of iron, and its proportion in Fe_2_O_3_ is 76.1%. After the introduction of heteroatoms, the Ra value of Fe_2_O_3_ catalyst is much greater than that of pure Fe_2_O_3_ (0.339), revealing the existence of Ni in the lattice of Fe_2_O_3_, replacing Fe^2+^ in the form of Ni^2+^. Associated with XRD results, Ni tends to replace Fe to form the structure of NiFe_2_O_4_ on the catalyst surface, which also explains why the principal valence state of Ni is +2 [29]. In general, the presence of Ni^2+^ ions are concomitant with the formation of O_Vs_, which signally affects the catalytic performance. The introduced heteroatoms indeed boost the formation of O_Vs_. At the same time, the composition of Ni^3+^-O_Vs_-Fe^2+^ can constitute an active site to promote the oxidation of soot [9]. After the introduction of surface defects, as shown in Figure S6, the fraction of Ni^2+^ ions increased from 15% to 25%. Additionally, as shown in Figure 4C,D, the content of O_A_ showed a trend of decreasing after rising, and reached the maximum in Ni-Fe_2_O_3_-20 (0.27), indicating that the surface O_Vs_ increased and the oxidation capacity was enhanced. On the one hand, it was conducive to the adsorption of O_2_. On the other hand, it contributed to the activation of O_2_.

### 3.5. H_2_-TPR Profiles

**Figure 5:**
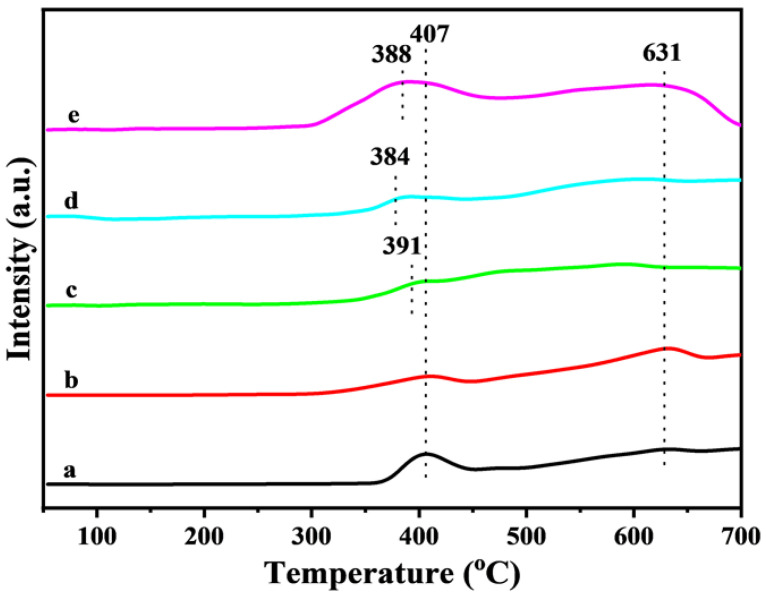
H_2_-TPR profiles of Fe_2_O_3_ and Ni−Fe_2_O_3_-X catalysts: (a) Fe_2_O_3_, (b) Ni-Fe_2_O_3_-5, (c) Ni-Fe_2_O_3_-10, (d) Ni-Fe_2_O_3_-20, (e) Ni-Fe_2_O_3_-30.

The oxygen vacancy generated in the catalyst satisfies the condition of changing the redox state of the catalyst so as to achieve equilibrium with the actual gas-phase oxygen. This result was confirmed by H_2_-TPR [30]. The results are presented in Figure 5. Fe_2_O_3_ catalysts have two main peaks at 407 °C and 631 °C, due to the gradual reduction of Fe_2_O_3_ to Fe_3_O_4_ and eventually from Fe_3_O_4_ to FeO. H_2_-TPR of pure NiO is shown in Figure S9. A clear reduction peak can be seen at 391 °C. This reduction peak curve is generally considered to be the reduction of Ni^2+^ to the metallic-state Ni. When Ni ions partially replace Fe ions at 407 °C, all the reduction peak temperatures of the catalyst shift to the low-temperature direction (388 °C). The most important reason for this is that as the addition of Ni gradually increases, due to the electron donor effect of Ni ions, the electron cloud density of A-site ions is transferred to B-site ions, so that the electron ability of Fe^3+^ is improved, and it is easier to be reduced to Fe^2+^. However, compared with Ni-Fe_2_O_3_-20, although the content of Ni ions in Ni-Fe_2_O_3_-30 is increased, the position of the reduction peak is lower than that of the former. A possible reason for this is that the Ni-Fe_2_O_3_-20 catalyst has a stronger Fe-Ni synergistic effect, and the formed NiFe_2_O_4_ structure is easier for Fe^3+^ to exchange electrons into Fe^2+^. In addition, in the soot combustion reaction, the active site of the catalyst promotes the oxidation of soot, and the actual reaction temperature is low, so the reduction peak in the low temperature range is the focus of catalytic soot oxidation reaction. It can be seen from Figure 5 that with the increase in Ni content, the reduction peak in the low-temperature section significantly widens the range of reduction temperature. Combined with the analysis of XPS results, this may be due to the charge compensation caused by partial Ni ion substitution, resulting in more chemisorbed oxygen on the catalyst surface. In addition, Ni-Fe_2_O_3_-20 < Ni-Fe_2_O_3_-30 < Ni-Fe_2_O_3_-10 < Ni-Fe_2_O_3_-5 for the reduction peak temperature at low temperature, and Ni-Fe_2_O_3_-20 catalyst also has the highest H_2_ oxidation amount. These results indicate that an appropriate amount of Ni doping can improve the reducing ability and surface-active oxygen density of the catalyst. Therefore, the Ni-Fe_2_O_3_-20 catalyst has the best low-temperature reduction capacity, abundant reactive oxygen species and high oxygen mobility, which matches the XPS, TOF and activity results. Therefore, the above experiments can verify that the escalation of adsorption and activation of O_2_ on the Fe_2_O_3_ surface is due to the synergistic effect of Fe-Ni [31]. The strong adsorption ensures the rapid renewal of surface oxygen in a relatively oxygen-rich environment, showing a strong oxidation competence. The strong adsorption caused by this synergistic effect maintained the rapid renewal of the oxygen which is on the surface of the catalyst, and the oxidation capacity was also maintained at a strong level.

### 3.6. Catalytic Performance

**Figure 6:**
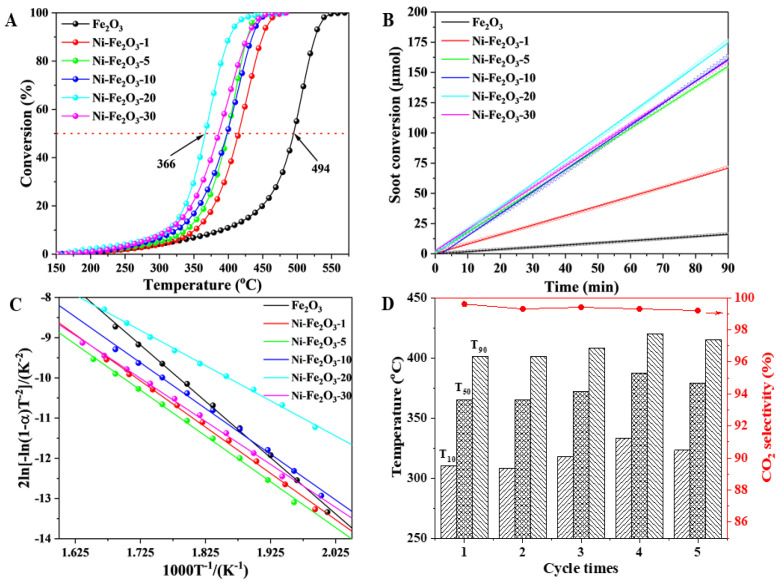
(**A**) Soot-TPO tests; (**B**) cumulative conversion amounts of soot particles during isothermal oxidation reaction of 300 °C; (**C**) Ozawa plots for soot conversion of 50% over Fe_2_O_3_ and Ni−Fe_2_O_3_-X catalysts; (**D**) the cycle stability of Ni-Fe_2_O_3_-20 catalysts [33].

The catalytic oxidation performance of Fe_2_O_3_ and Ni-Fe_2_O_3_-X catalysts in the process of soot combustion was tested. The contact mode between catalyst and soot adopts the loose contact mode as described above. The results are shown in Figure 6A and Table 2. When tested in pure soot without catalyst, the T_50_ is 585 °C, and the SCO_2_^m^ is only 65.2%. As shown in Figure S3, compared with common Fe_2_O_3_ (T_50_ = 488 °C), Fe_2_O_3_ prepared in this experiment (T_50_ = 474 °C) has certain advantages. After doping Ni into the Fe_2_O_3_ catalyst, it can be clearly observed from Figure 6A that the conversion curve of soot moves from 494 °C towards a low temperature (366 °C). The drop is 128 °C, indicating that the catalytic oxidation efficiency is significantly improved by the catalyst. As shown in Table 2, compared with a pure Fe_2_O_3_ or NiO catalyst, the soot catalytic activity and CO_2_ selectivity of Ni-Fe_2_O_3_-X catalysts are significantly improved. As shown in Figure S7, the T_50_ value of NiO is 437 °C, which exclude the contribution of the pure NiO catalyst to soot oxidation. By comparison, the T_50_ value of Ni-Fe_2_O_3_ catalysts can be reduced by at least 170 °C on the basis of pure soot combustion, and the selectivity of CO_2_ is always close to 100%, which indicates that the incorporation of Ni into the Fe_2_O_3_ catalyst as the active site is of importance in the oxidation efficiency of soot. Among all catalysts, the catalytic oxidation activity of Ni-Fe_2_O_3_-20 on soot is the best; T_50_ is 366 °C, and the value of SCO_2_^m^ is 99.1%, close to 100%. With the enhancement of Ni content, the catalytic oxidation activity of soot decreased. It can be seen from XRD that the NiFe_2_O_4_ crystal phase on the surface weakens with the increase in Ni incorporation, indicating that the synergistic effect of Ni-Fe also decreases gradually, which is consistent with the change in activity. As shown in Table 1, taking Pt/Fe_2_O_3_ as an example [7], it can be found that T_10_, T_50_ and T_90_ (297, 365, 418 °C) of the catalyst are all at a low temperature after adding Pt into Fe_2_O_3_ by the dipping method. However, the Ni-Fe_2_O_3_-20 catalyst does not introduce any noble metals, and the T_50_ value is similar to Pt/Fe_2_O_3_. This kind of catalyst, without blending noble metals, has higher economic benefits, and these metals are easy to obtain which brings great help to future research and exploration.

In the process of understanding the intrinsic properties of the catalyst, TOF can accurately react to it. Influenced by the kinetic regime, the test temperature of TOF was set at 300 °C. Ro is defined as the ratio of reaction rate values (μmol g^− 1^min^− 1^), which is calculated by fitting the slope of the cumulative CO_x_ quantity (μmol g^−1^) to the reaction time (min) curve. At the same time, the O* quantity can be determined in the same way. Unlike the process for determining Ro, there is no O_2_ in the process for determining the amount of O*. The Fe_2_O_3_ catalyst’s reaction rate demonstrates the least ideal situation at 300 °C, the value of which is 4.4 μmol g^−1^ min^−1^. Compared with the former, Ni-Fe_2_O_3_-X catalysts display a higher reaction rate during soot purification. Particularly, Ni-Fe_2_O_3_-20 catalysts reveal a reaction rate of 21.0 μmol g^−1^ min^−1^. Rate is also correlated with activity. It can be seen from the above that this catalyst also has the best activity, with a T_10_ of 310 °C. Conversely, active oxygen (O* amount) is also an important parameter affecting the TOF value, which is determined by isothermal anaerobic titration. The O* quantity can be obtained by calculation, and the results are summarized in Table 2. By comparison, it can be found that the O* amount of the catalysts matches the result of the Ro value. In the midst of all catalysts, the O* amount of Fe_2_O_3_ catalyst is the lowest value at 79.6 μmol g^−1^. From the changing trend of the lines after the introduction of Ni in the picture, it can be clearly observed that the O* density exhibits a slight increase. Among them, the Ni-Fe_2_O_3_-20 catalyst’s O* density is 134.0 μmol g^−1^. It outperforms the other catalysts by a surprising margin. This result, compared to the worst Fe_2_O_3_ catalyst, is almost a 1.7-fold increase. Herein, the value of TOF over Fe_2_O_3_ and Ni-Fe_2_O_3_-X catalysts can be calculated via isothermal reactions, and is gathered in Table 1. The same conclusion is also shown in Figure S1. Based on the results of TOF value, it is noted that the introduced Ni provided extra active sites to enhance the adsorption–activation capacity for gaseous O_2_, boosting the removal of soot particles. Among all, the Ni-Fe_2_O_3_-20 catalyst presents the highest TOF value (0.156 h^−1^ at 300 °C). The conclusion can be proved by the consequence that Ni-Fe_2_O_3_-20 has the best intrinsic activity. As another important parameter to evaluate the intrinsic activity, the apparent activation energy (Ea) represents the ease of the reaction. This performance affects the frequency of soot conversion. The method commonly used to calculate Ea is the Ozawa method. The calculated results are shown in Figure 6C and Table 2. The Ea value of the Fe_2_O_3_ catalyst is 116.4 kJ mol^−1^. With the substitution of Fe atoms by Ni atoms, the Ni-Fe_2_O_3_-20 catalyst presents the lowest Ea value (72.7 kJ mol^−1^). The results correspond to the result of the trend of TOF values, and are similar to the ignition activity [32]. Thus, the Ni-Fe_2_O_3_-X catalyst can perform the best elimination efficiency. In practical applications, it is also necessary to consider the cyclic stability of the catalyst. This performance determines whether the catalyst can be reused to achieve cost savings. It is known that the Ni-Fe_2_O_3_-20 catalyst has the best catalytic performance among all catalysts, so this catalyst was selected to carry out the same soot-TPO test four times. As shown in Figure 6D, the values of T_10_, T_50_ and T_90_ almost keep in a certain range. Moreover, after four cycles, the SCO_2_^m^ also remains at a high level. The value is 99.1%. This phenomenon implies its excellent stability for catalytic soot purification. The hexagonal nanosheet retains its original shape after four cycles (Figure S2) and other particles were not observed on the surface of Fe_2_O_3_. By analyzing this representation, the following conclusions that Ni atoms have been successfully introduced into Fe_2_O_3_ to form a more stable structure can be drawn. Such non-noble-metal catalysts are cheap, readily available and highly effective in eliminating the catalytic activity of soot, which renders them promising candidates for soot removal, especially the Ni-Fe_2_O_3_-20 catalyst. Additionally, as shown in Figure S2, TEM and HRTEM images of the Ni-Fe_2_O_3_-uesd catalyst indicates that the catalyst presents a polyhedral structure after use. This also proves the stability of the catalysts.

### 3.7. Surface Chemical State of Ni-Fe_2_O_3_-X Catalysts

**Figure 7:**
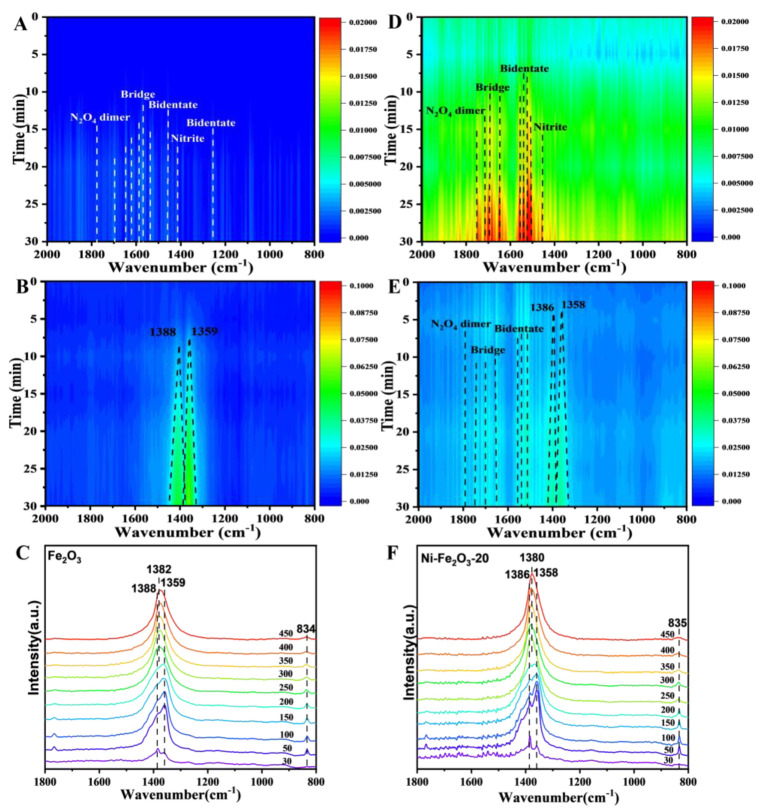
NO adsorption and oxidation at 30 °C in situ DRIFTS results of (**A**,**B**) Fe_2_O_3_ and (**D**,**E**) Ni-Fe_2_O_3_-20; NO oxidation in situ DRIFTS results of the (**C**) Fe_2_O_3_ and (**F**) Ni-Fe_2_O_3_-20 catalysts.

It is a prerequisite to determine the synergistic effect of Fe and Ni to clarify the oxidation reaction pathway of soot. As can be seen from Figure 7A and Figure S8, the Ni-Fe_2_O_3_-20 catalyst’s catalytic performance is T_50_ = 366 °C. The air condition includes 40 ppm NO and the catalyst is in loose contact with the soot. The identical catalysts without NO have a noticeable drop in performance, and the activity is decreased by 22 °C and the CO_2_ selectivity is decreased by 8.3% as well due to the strong oxidation and migration capacity of the NO_2_ formed. These results indicate that NO_2_-assisted oxidation exhibits a leading role in catalytic soot formation [30]. The relevant equations are as follows:

C_soot_ + NO_2_ → C(O) + NO (1)

C_soot_ + Fe^x+^ − NO_3_ → C(O) + Fe··x+ − O + NO (2)

**Figure 8:**
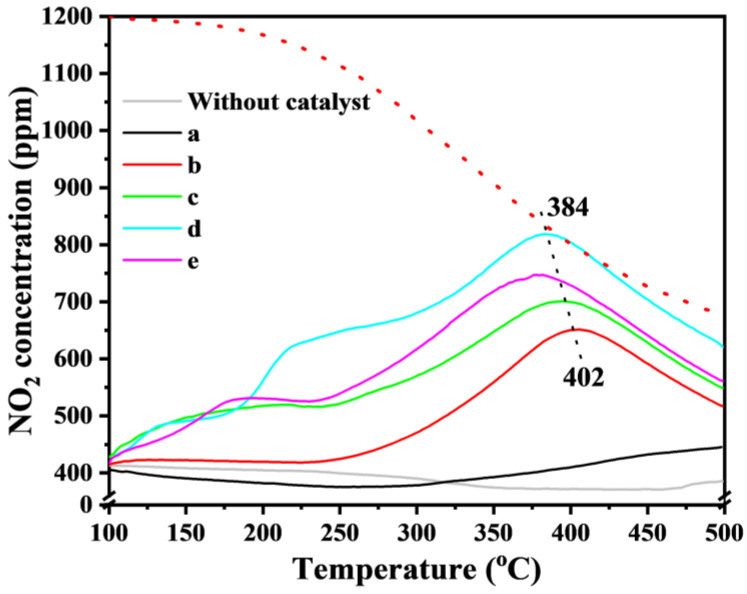
NO_2_ concentration curves of NO temperature-programmed oxidation over Fe_2_O_3_ and Ni−Fe_2_O_3_-X catalysts: (a) Fe_2_O_3_, (b) Ni-Fe_2_O_3_-5, (c) Ni-Fe_2_O_3_-10, (d) Ni-Fe_2_O_3_-20, (e) Ni-Fe_2_O_3_-30.

NO_2_ acts as a powerful transfer agent of reactive oxygen species. The higher the NO_2_ fraction yield, the higher the reaction rate. The surface intermediates formed by Ni-Fe_2_O_3_-X during the oxidation of NO are an indispensable part of studying the effect of nitrogen oxides on the catalyst surface. In situ DRIFT testing and NO-TPO provide insight into and analysis of this process. It can be seen from the comparison between Figure 7A and Figure 7D that the Ni-Fe_2_O_3_-20 catalyst is more likely to adsorb NO after the introduction of NO, and exists in the form of nitrate on the catalyst surface. However, the adsorption strength of pure Fe_2_O_3_ is relatively small. After the introduction of O_2_, only about 1400 cm^−1^ of the nitrate formed was retained on pure Fe_2_O_3_. On the Ni-Fe_2_O_3_-20 catalyst, the nitrite strength is higher and more stable (Figure 7B,E). Therefore, through the synergistic effect between Ni-Fe, the Ni-Fe_2_O_3_-20 catalyst can stably maintain a certain amount of surface nitrite, without being affected by external O_2_, so as to better store NO_2_ as surface nitrate. This result proves that NO_2_-assisted oxidation plays a leading role in the catalytic process of soot. In situ DRIFTS were tested at a 30 °C temperature gradient between 30 °C and 450 °C. As shown in Figure 7C, the peaks of Fe_2_O_3_ catalyst are nitrate (1388 cm^−1^ and 1359 cm^−1^). With the increase in temperature, the nitrite gradually changes to monodentate nitrate (1380 cm^−1^), and reached its maximum intensity at 400 °C, which was consistent with the highest fraction yield of NO_2_ [31]. In Figure 7F, NO_x_ on the surface of the Ni-Fe_2_O_3_-20 catalyst is mainly in the form of nitrite (1386 and 1358 cm^−1^). By comparing the two catalysts, it can be found that the peak position of the two catalysts does not change with the increase in temperature, indicating that the desorption of NO_2_ is not the rate-determining step. Stable free NO_2_- gradually disappears with increasing temperature and gradually merges into monodentate nitrate at 300 °C (1380 cm^−1^). In the NO-TPO test, the Ni-Fe_2_O_3_-X catalyst generated NO_2_ molecules more efficiently than the Fe_2_O3 catalyst (Figure 8). By comparing the above results of Fe2O3 and Ni-Fe2O3-x catalysts, it is confirmed that another key role of Ni ions is to reduce the adsorption of NOx, which leads to the increase in NO_2_ molecules in the NO-TPO test. Combined with DRIFTS results and catalytic performance, it can be seen that NO_2_-assisted oxidation plays a leading role in the formation of catalytic soot, and Ni-Fe_2_O_3_-X catalyst significantly promotes the renewal of active oxygen species and the desorption of NO_2_ molecules, showing the best performance in catalytic dust purification.

## 4. Conclusions

In summary, we have developed a highly active and stable Ni-Fe_2_O_3_-X catalyst. The structure of NiFe_2_O_4_ is formed on the surface of Fe_2_O_3_ nanoparticles through a Fe-Ni synergism, which enhances the interaction between the Ni and Fe_2_O_3_- {110} carrier, promotes the formation of coordination unsaturated ions of reactants, generates more oxygen vacancies thus promoting the adsorption of NO and accelerates the low-temperature ignition of soot in O_2_ [34]. The results show that the synergic effect of Fe-Ni ensures the excellent catalytic activity of Ni-Fe_2_O_3_-X catalyst (T50 = 366 °C, TOF = 0.156 min^−1^, Ea = 72.7 kJ mol^−1^) during soot oxidation. In addition, the superior catalytic properties were maintained well after five cycles, making the catalyst a promising candidate for future applications. This study reveals the key role of intermetallic synergies in catalyst surface modification, and provides a promising strategy for further development of efficient and low-cost catalysts for automotive exhaust soot removal without precious metals.

## Figures and Tables

**Table 1:** Prepared catalyst and Fe/Ni ratios.

Sample	Fe (NO_3_)_3_·9H_2_O (mmol)	Ni (NO_3_)_2_·6H_2_O (mmol)
Fe_2_O_3_	5.0	0
Ni-Fe_2_O_3_-1	4.95	0.05
Ni-Fe_2_O_3_-5	4.75	0.25
Ni-Fe_2_O_3_-10	4.5	0.5
Ni-Fe_2_O_3_-20	4.0	1.0
Ni-Fe_2_O_3_-30	3.5	1.5

**Table 2:** All catalytic values of Fe_2_O_3_ and Ni−Fe_2_O_3_-X catalysts for soot removal by loose contact.

	T_10_(^o^C)	T_50_(^o^C)	T_90_(^o^C)	Sco_2_^m^ (%)	R (μmol g^−1^ min^−1^)	O* Amount (μmol g^−1^)	TOF (min^−1^)	H_2_ Consumption (μmol g^−1^)	Ea (kJ mol^−1^)
Soot	461	584	648	65.2	-	-	-	-	-
Fe_2_O_3_	390	494	526	84.1	4.4	79.6	0.055	959.7	116.4
Ni-Fe_2_O_3_-1	351	414	450	98.4	12.1	89.4	0.135	1118.6	95.6
Ni-Fe_2_O_3_-5	338	398	427	96.0	17.3	121.4	0.142	979.3	94.7
Ni-Fe_2_O_3_-10	324	398	434	99.4	18.7	126.6	0.147	1417.0	94.5
Ni-Fe_2_O_3_-20	310	366	402	99.1	21.0	134.0	0.156	1144.9	72.7
Ni-Fe_2_O_3_-30	312	386	429	99.7	17.9	136.2	0.143	1692.7	88.9
Pt_2_/Fe_2_O_3_	297	365	418	98.8	22.5	136.8	0.164	2233.7	69.7

## Data Availability

Data can be obtained from the contact author on request.

## Supplementary Materials

The following are available online at https://www.mdpi.com/article/10.3390/nano15030233/s1, Detail of Materials Characterization and Catalytic Performance Evaluation, Figure S1: The CO_2_ concentration curves at 310 °C as a function of time over catalysts under anaerobic condition: (A) Fe_2_O_3_; (B) Ni-Fe_2_O_3_-1; (C) Ni-Fe_2_O_3_-5; (D) Ni-Fe_2_O_3_-10; (E) Ni-Fe_2_O_3_-20; (F) Ni-Fe_2_O_3_-30, Figure S2: TEM and HRTEM images of uesd Ni-Fe_2_O_3_-20 catalysts, Figure S3: The catalytic performance of common Fe_2_O_3_ and Fe_2_O_3_ nanosheets (this work) for soot oxidation at same condition, Figure S4: The XRD patterns of fresh Ni-Fe_2_O_3_-20 and uesd Ni-Fe_2_O_3_-20 catlysts, Figure S5: Ni 2p spectra of Ni-Fe_2_O_3_ catalysts, Figure S6: Ni, Fe percentage content of (a) Fe_2_O_3_; (b) Ni-Fe_2_O_3_-5; (c) Ni-Fe_2_O_3_-10; (d) Ni-Fe_2_O_3_-20; (e) Ni-Fe_2_O_3_-30 catalysts, Figure S7: soot-TPO test of NiO, Figure S8: soot-TPO test of Ni-Fe_2_O_3_-20 catalyst without NO, Figure S9: The XRD patterns of NiO, Table S1: The elemental composition of as-prepared catalysts obtained by ICP-OES, Table S2: Surface elemental composition and valance states of Fe (2p) and O (1s) species over as-prepared Fe_2_O_3_ and Ni-Fe_2_O_3_-X catalysts derived from XPS analysis.
