# Supplementary material for: Efficient Purification of Auto-Exhaust Soot Particles Using Hexagonal Fe2O3 Nanosheets Decorated with Non-Noble Metals (Ni)
Source: Nanomaterials (Basel). 2025 Feb 1;15(3):233. doi: 10.3390/nano15030233 (PMC11820007; doi:10.3390/nano15030233)
Supplement: Supplementary file 1 [file nanomaterials-15-00233-s001.zip › nanomaterials-3451694-supplementary.pdf]

Supplementary Materials

Efficient Purification of Auto-Exhaust Soot Particles using Hexagonal Fe_2O_3 Nanosheets Decorated with Non-Noble Metals (Ni)

Haoqi Guo [1,†], Jing Xiong [1,2,†,*], Peng Zhang [1,†], Jian Liu [1], Zhen Zhao [1] and Yuechang Wei [1,2,*]

[1] State Key Laboratory of Heavy Oil Processing, College of Science, China University of Petroleum, Beijing 102249, China; 2022211354@student.cup.edu.cn (H.G.), 2019310807@student.cup.edu.cn (P.Z.), liujian@cup.edu.cn (J.L.), zhenzhao@cup.edu.cn (Z.Z.)

[2] Key Laboratory of Optical Detection Technology for Oil and Gas, China University of Petroleum, Beijing 102249, China

* Correspondence: Correspondence: xiongjing@cup.edu.cn (J.X.), weiyc@cup.edu.cn (Y.W.)

† These authors contributed equally to this work.

1. Supplementary Sections

1.1. Materials Characterization

The Powder X-ray diffraction (XRD) measurements were performed using the Bruker D8 Advance (Bruker Axs Gmbh, Germany) X-ray diffractometer (λ = 1.5406 A) with a 2θ Angle of 5°~ 90° and a scanning rate of 5°min^{-1}. Raman spectra were measured in the inverse Stokes range from 200 to 1300 cm^{-1} with the InVia Reflex Ranisho spectrometer of Renishaw, UK, and samples were excited with a 532 nm He-Ge laser. Scanning electron microscopy (SEM, FEI Quanta 200F, FEI Company, Eindhoven, Holland) and transmission electron microscopy (TEM, JEOL JEM 2100, JEOL Company) were used. The morphology and microstructure of the Ni-Fe_2O_3-20 catalyst were studied. The surface properties of Fe_2O_3 and Ni-Fe_2O_3-X catalysts were studied by Perkin-Elmer PHI-1600 ESCA X-ray photoelectron spectrometer (XPS, Massachusetts, USA) using a monochromatic mg-k-α X-ray source. The hydrogen consumption experiment of H_2-temperature programmed reduction (H_2-TPR) was measured by Autosorb IQ Quantachrome, State of California, USA. NO programmed oxidation (NO-TPO) was performed in a fixed bed reactor. The catalyst (0.1g) was pretreated with N_2 at 100 °C for 30 min and then heated from 100 °C to 450 °C at 2 °C min^{-1}. At 50ml min^{-1}, NO (0.2 vol %), O_2 (5 vol %), and N_2 were used as equilibrium atmospheres. In situ diffuse infrared Fourier transform spectroscopy (DRIFTS) has been measured on an IR Tracer-100 spectrometer equipped with a liquid nitrogen-cooled MCT detector. The catalyst was pretreated in an N_2 atmosphere. The temperature is 200 °C. The heating time is one hour. And then cooled the catalyst to 50°C to collect the background. The experiment was executed in an environmental reaction containing NO (0.2 vol %) and O_2 (5 vol %) in equilibrium with N_2 at a flow rate of 50 mL min^{-1}. Spectra are recorded to track changes in the surface material in the 50°C range from 50 °C to 400 °C.

1.1. Catalytic Performance Evaluation

The gas reactant containing O_2 (5%), NO (0.2%), and Ar as equilibrium gas proceeded through the mixture of catalyst and soot. The gas reactant consisting of O_2 (5%), NO (0.2%), and Ar as equilibrium gas proceeded through the mixture of catalyst and soot. The flow rate of this gas mixture is 50 mL min^{-1}. On-line Gas chromatography Analysis with FID detector for export gas products (GC 9890, Shanghai Star Analytical Instruments Co., LTD., Shanghai). The catalytic soot combustion activity was estimated by the values of T_{10}, T_{50}, and T_{90}, which were defined as the temperatures at 10%, 50%, and 90% of soot

conversion, respectively. The selectivity of generated gas to CO_2 (SCO_2) was defined by the equation: $SCO_2 = CCO_2/\ (CCO + CCO_2)$, and SCO_2^m was defined as SCO_2 at the maximum of CCO_2. The turnover frequency (TOF) of a constitutive active catalyst is defined as the ratio of isothermal reaction rate (R) to reactive oxygen species density (Do) at the active site. The oxidation isothermal reaction rate of dust was obtained under the condition of stability and low conversion of soot particles. At 300°C, the conversion rate of soot can only be maintained below 10%, and the reaction rate hardly changes, so isothermal oxidation is carried out at this temperature. Since the conversion rates of soot were low (<10%) and the reaction rates were almost steady, the isothermal oxidation of soot was carried out at 300°C. At 300°C, the surface density of the active site of catalyst reaction with soot can be determined by isothermal anaerobic titration.

2. Supplementary Figures

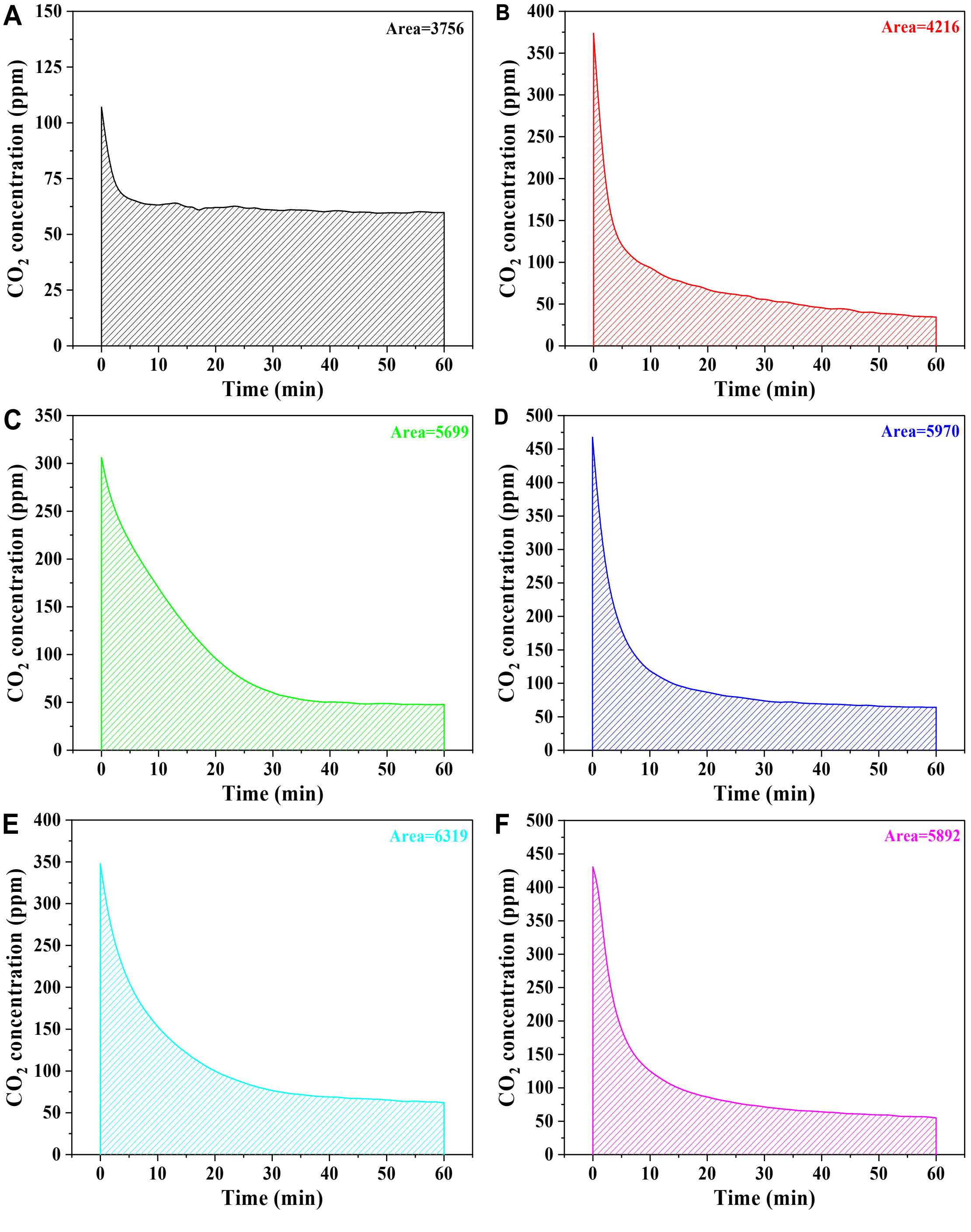

Figure S1. The CO_2 concentration curves at 310°C as a function of time over catalysts under anaerobic conditions: (A) Fe_2O_3; (B) Ni-Fe_2O_3-1; (C) Ni-Fe_2O_3-5; (D) Ni-Fe_2O_3-10; (E) Ni-Fe_2O_3-20; (F) Ni-Fe_2O_3-30.

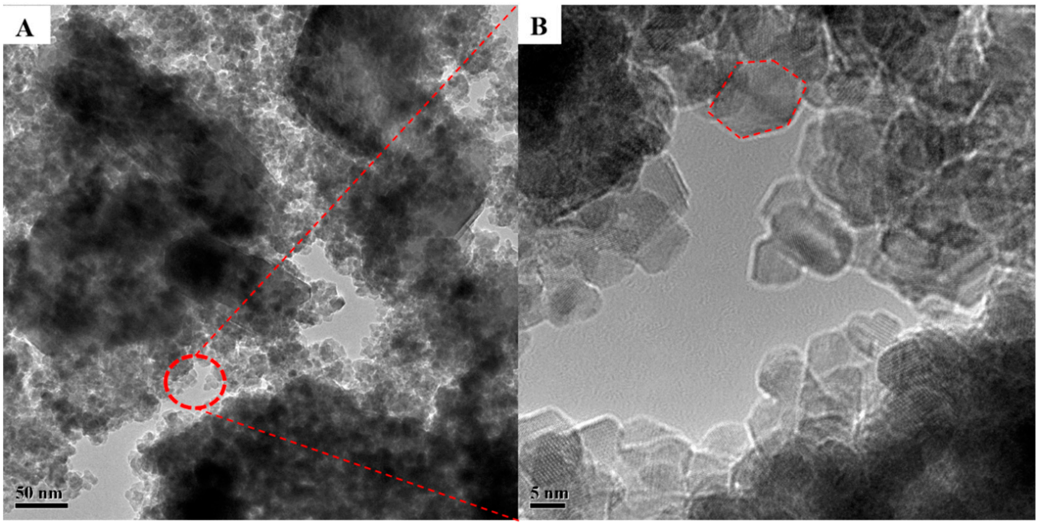

Figure S2. TEM and HRTEM images of used Ni-Fe_2O_3-20 catalysts catalyst.

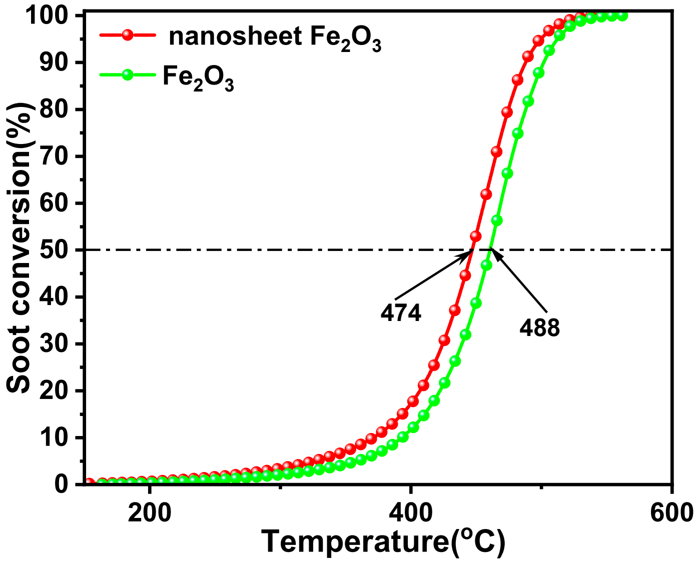

Figure S3. The catalytic performance of common Fe_2O_3 and Fe_2O_3 nanosheets (this work) for soot oxidation at the same condition.

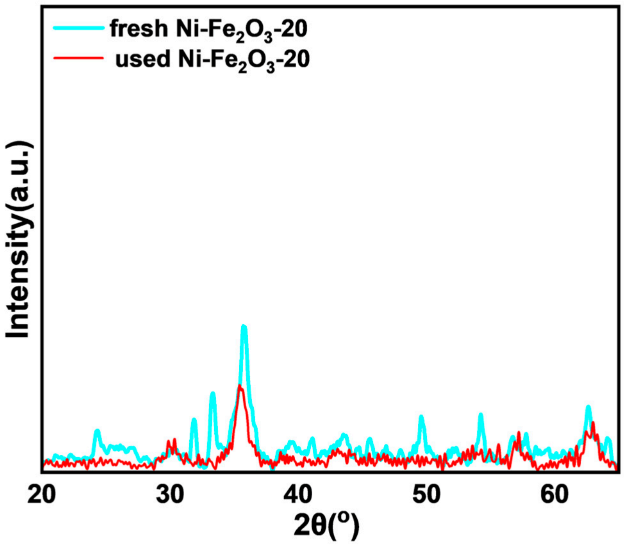

Figure S4. The XRD patterns of fresh Ni-Fe_2O_3-20 and used Ni-Fe_2O_3-20 catalysts.

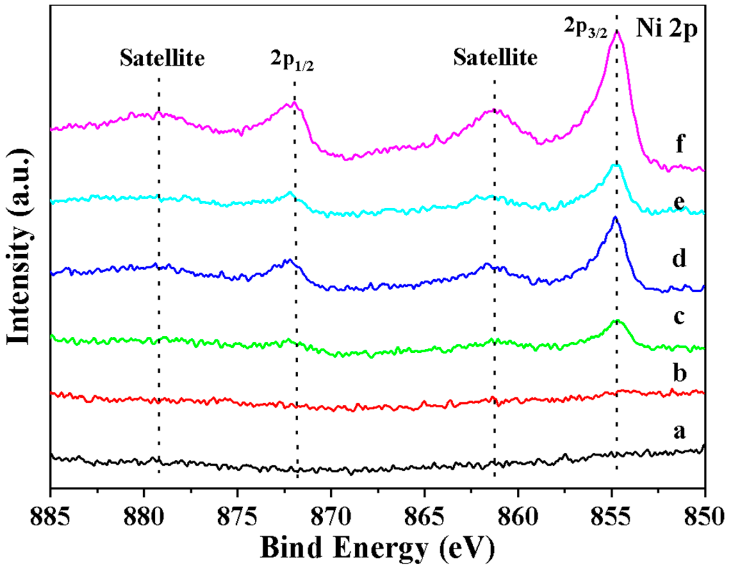

Figure S5. Ni 2p spectra of Ni-Fe_2O_3-X catalysts.

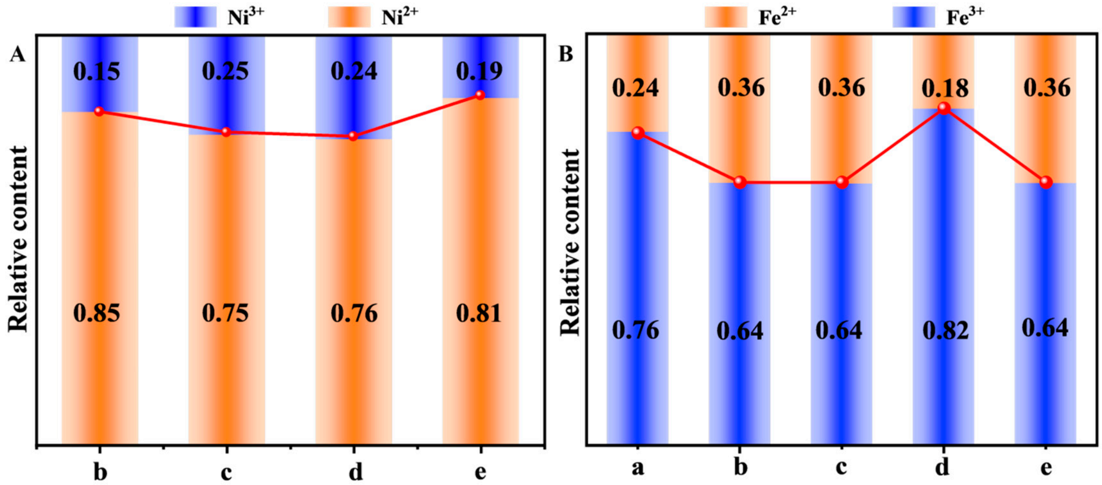

Figure S6. Ni, Fe percentage content of (a) Fe_2O_3; (b) Ni-Fe_2O_3-5; (c) Ni-Fe_2O_3-10; (d) Ni-Fe_2O_3-20; (e) Ni-Fe_2O_3-30 catalysts.

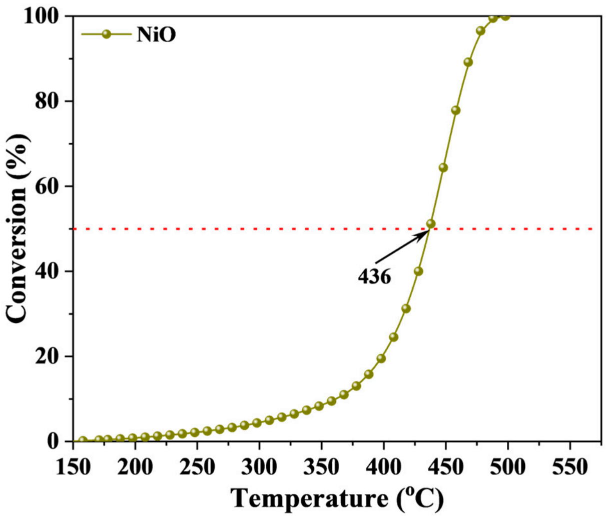

Figure S7. soot-TPO test of NiO.

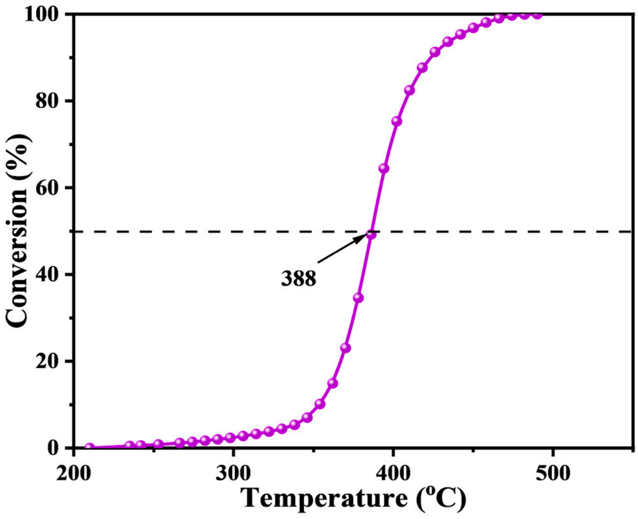

Figure S8. soot-TPO test of Ni-Fe_2O_3-20 catalyst without NO.

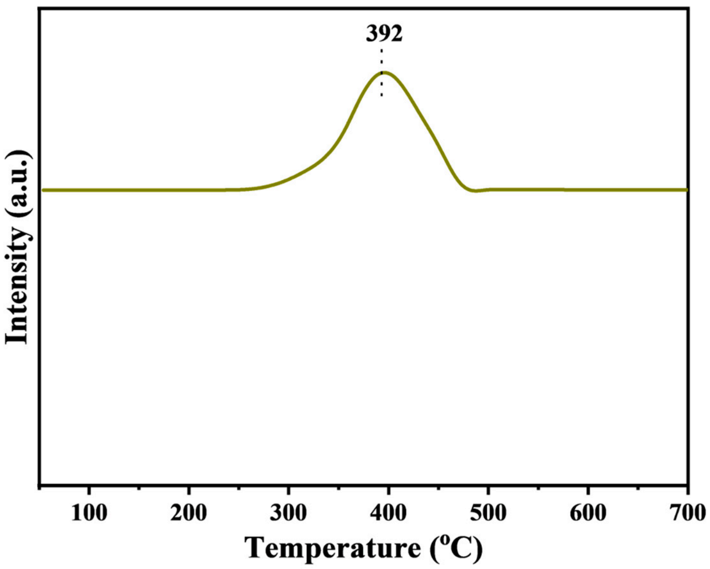

Figure S9. The XRD patterns of NiO.

3. Supplementary Tables

Table S1. The elemental composition of as-prepared catalysts obtained by ICP-OES.

Catalysts	Ni(%)	Fe(%)
Ni-Fe_2O_3-5	2.16	37.96
Ni-Fe_2O_3-10	4.21	35.78
Ni-Fe_2O_3-20	7.07	31.58
Ni-Fe_2O_3-30	13.11	27.91

Table S2. Surface elemental composition and valance states of Fe (2p) and O (1s) species over as-prepared Fe2O3 and Ni-Fe2O3 catalysts derived from XPS analysis.

Catalysts	Ni/Fe atomic ratio (%)	Fe species (%)			O species (%)		
		Fe^{3+}	Fe^{2+}	R^a	O_A	O_L	R^b
Fe_2O_3	-	84.7	15.3	0.181	29.3	70.7	0.414
Ni-Fe_2O_3-5	5.7	74.6	25.4	0.340	15.7	84.3	0.186
Ni-Fe_2O_3-10	11.8	75.7	24.3	0.321	19.8	80.2	0.247
Ni-Fe_2O_3-20	22.4	81.3	18.7	0.230	20.1	79.9	0.252
Ni-Fe_2O_3-30	47.0	76.1	23.9	0.314	19.7	80.3	0.246
NiO	-	-	-	-	24.2	75.8	0.319

[a] The relative ratio of Fe^{2+}/Fe^{3+}

[b] The relative ratio of the surface adsorbed oxygen O_A (O_2^{2-} and O_2^-) to lattice oxygen O_L (O^{2-}).

References

[1] P. Legutko, M. Fedyna, J. Gryboś, X. Yu, Z. Zhao, A. Adamski, A. Kotarba, Z. Sojka, Intricate role of doping with d^0 ions (Zr^{4+}, V^{5+}, Mo^{6+}, W^{6+}) on cryptomelane (K-OMS-2) performance in the catalytic soot combustion in presence of NO and SO_2, *Fuel*. **2022**, *328*, 125325. https://doi.org/10.1016/j.fuel.2022.125325.

[2] P. Zhang, J. Xiong, Y. Wei, Y. Li, Y. Zhang, J. Tang, W. Song, Z. Zhao, J. Liu, Exposed {001} facet of anatase TiO_2 nanocrystals in Ag/TiO_2 catalysts for boosting catalytic soot combustion: The facet-dependent activity, *J. Catal*. **2021**, *398*, 09–122. https://doi.org/10.1016/j.jcat.2021.04.015.

[3] P. Zhang, X. Mei, X. Zhao, J. Xiong, Y. Li, Z. Zhao, Y. Wei, Boosting catalytic purification of soot particles over double perovskite-type $La_{2-x}K_xNiCoO_6$ catalysts with an ordered macroporous structure, *Environ. Sci. Technol*. **2021**, *55*, 11245–11254. https://doi.org/10.1021/acs.est.1c01781.

[4] Y. Yang, D. Zhao, Z. Gao, Y. Tian, T. Ding, J. Zhang, Z. Jiang, X. Li, Interface interaction induced oxygen activation of cactus-like Co_3O_4/OMS-2 nanorod catalysts in situ grown on monolithic cordierite for diesel soot combustion, *Appl. Catal. B*. **2021**, 286, 119932. https://doi.org/10.1016/j.apcatb.2021.119932.

[5] X. Yu, Y. Ren, D. Yu, M. Chen, L. Wang, R. Wang, X. Fan, Z. Zhao, K. Cheng, Y. Chen, J. Gryboś, A. Kotarba, Z. Sojka, Y. Wei, J. Liu, Hierarchical porous K-OMS-2/3DOM-m $Ti_{0.7}Si_{0.3}O_2$ catalysts for soot combustion: Easy preparation, high catalytic activity, and good resistance to H_2O and SO_2, *ACS Catal*. **2021**, *11*, 5554-5571. https://doi.org/10.1021/acscatal.1c00748.

[6] Y. Li, P. Zhang, J. Xiong, Y. Wei, H. Chi, Y. Zhang, K. Lai, Z. Zhao, J. Deng, Facilitating catalytic purification of auto-exhaust carbon particles via the Fe_2O_3{113} facet-dependent effect in Pt/Fe_2O_3 catalysts, *Environ. Sci. Technol*. **2021**, 55, 16153–16162. https://doi.org/10.1021/acs.est.1c05908.

[7] Q. Yu, J. Xiong, Z. Li, X. Mei, P. Zhang, Y. Zhang, Y. Wei, Z. Zhao, J. Liu, Optimal exposed crystal facets of α-Mn_2O_3 catalysts with enhancing catalytic performance for soot combustion, *Catal. Today*. **2021**, *376*, 229–238. https://doi.org/10.1016/j.cattod.2020.05.039.

[8] J. Xiong, Z. Li, P. Zhang, Q. Yu, K. Li, Y. Zhang, Z. Zhao, J. Liu, J. Li, Y. Wei, Optimized Pt-MnO_x interface in Pt-MnO_x/3DOM-Al_2O_3 catalysts for enhancing catalytic soot combustion, *Chin. Chem. Lett*. **2021**, *32*, 1447–1450. https://doi.org/10.1016/j.cclet.2020.10.014.

[9] I. Meza-Trujillo, A. Mary, P. Pietrzyk, Z. Sojka, E.M. Gaigneaux, Nature and role of Cu(II) species in doped C12A7 catalysts for soot oxidation, *Appl. Catal. B*. **2022**, *316*, 121604. https://doi.org/10.1016/j.apcatb.2022.121604.

[10] Y. Wei, Y. Zhang, P. Zhang, J. Xiong, X. Mei, Q. Yu, Z. Zhao, J. Liu, Boosting the removal of diesel soot particles by the optimal exposed crystal facet of CeO_2 in Au/CeO_2 catalysts, *Environ. Sci. Technol.* **2020**, *54*, 002–2011. https://doi.org/10.1021/acs.est.9b07013.
